# Celecoxib may be a viable treatment option for breast cancer patients not treated with chemotherapy

**DOI:** 10.3389/fonc.2022.958308

**Published:** 2022-09-13

**Authors:** Randall E. Harris, Judith A. Schwartzbaum

**Affiliations:** ^1^ College of Medicine, The Ohio State University Wexner Medical Center, Columbus, OH, United States; ^2^ College of Public Health, The Ohio State University Wexner Medical Center, Columbus, OH, United States

**Keywords:** breast cancer, celecoxib, cyclooxygenase, clinical trial, inflammation

## Introduction

The *Randomized European Celecoxib Trial* (REACT) was a phase 3, multicenter, double-blind, placebo-controlled randomized clinical trial evaluating the selective cyclooxygenase-2 (COX-2) inhibitor celecoxib (400 mg daily for 2 years) as adjuvant therapy for 2,639 patients with early-stage primary breast cancer (1). Celecoxib has shown efficacy in preclinical studies of breast cancer and also as a chemopreventive agent in epidemiological investigations (2) and is currently under consideration as a repurposed drug in oncology as defined by the ReDO project “that seeks to repurpose well-known and well-characterized non-cancer drugs for new uses in oncology.” (3)

Based on their findings, the REACT investigators concluded that “patients showed no evidence of a disease-free survival (DFS) benefit for 2 years’ treatment with celecoxib compared with placebo as adjuvant treatment of ERBB2-negative breast cancer.” This interpretation omits an important observed benefit of celecoxib in a subgroup of patients who did not receive chemotherapy.

## Methods and results

**Figure 1:**
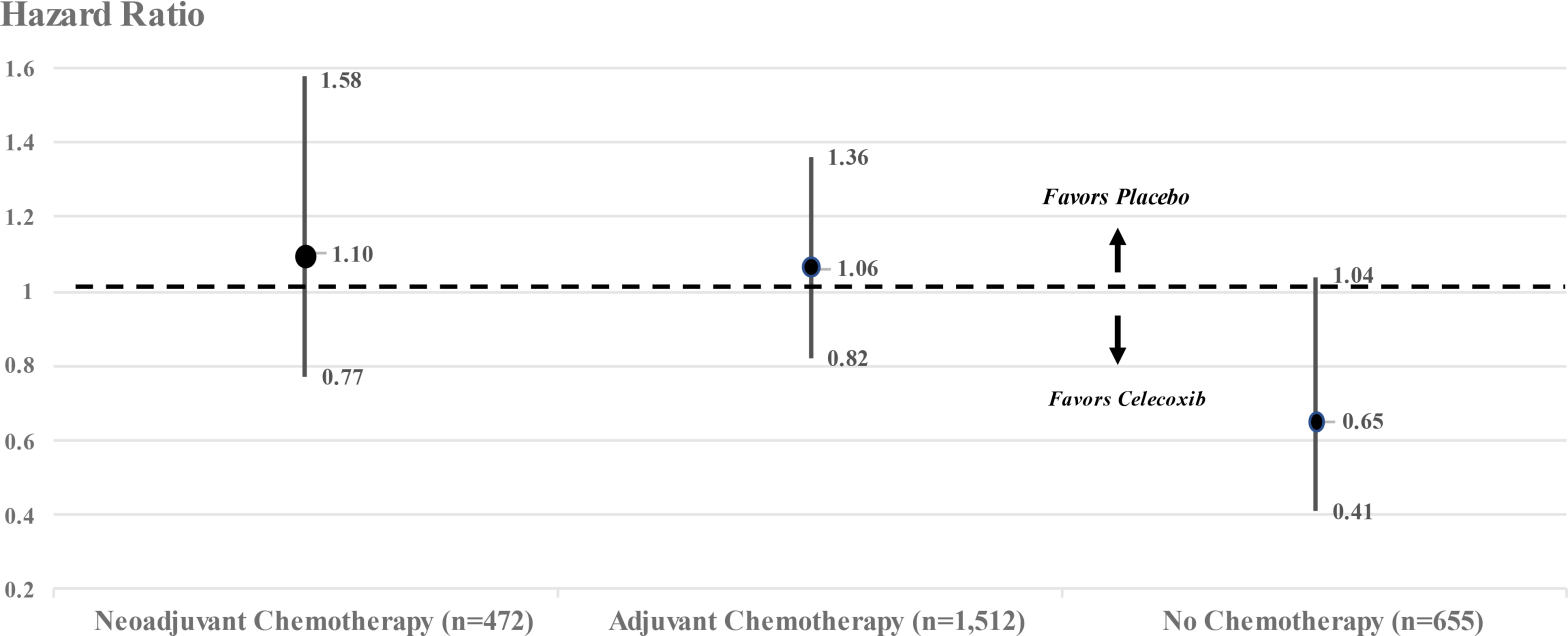
Hazard ratios and 95% confidence intervals for breast cancer patients stratified by chemotherapy in REACT. Adapted from Supplementary Online Content,eFigure: (1).

Though not discussed in the report, the REACT investigators did estimate that among 655 breast cancer patients who did not receive either adjuvant or neoadjuvant chemotherapy, treatment with celecoxib reduced the hazard ratio for recurrence over 10 years by 35% (HR = 0.65, 95% confidence interval 0.41–1.04) (**Figure 1**). Based on the *P*-values calculated from the confidence interval (4), this estimate barely missed statistical significance by a two-tailed test (*P* = 0.07) but did reach statistical significance by a one-tailed test (*P* = 0.035), which is the more important test since the trial was designed “to determine whether adjuvant celecoxib could improve disease outcomes among women with early breast cancer”.

## Discussion

The use of two-sided versus one-side tests of significance in scientific investigations, including human clinical trials, is a topic that has come under intense debate (5). Our purpose is not to dispel arguments on either side of using a one-sided versus a two-sided alternative hypothesis, but rather to call attention to the dismissal of a promising beneficial effect in view of the evidence to the contrary. In REACT, since the entire focus of the report is on the apparent absence of any significant benefit of celecoxib (1), we suggest that the use of a one-tailed test of significance is justifiable since the investigators assume that if celecoxib performed no better than placebo, then it would not be recommended as adjuvant therapy for ERRB-2-negative breast cancer. In other words, if treatment would only be altered if there is a significant benefit as opposed to no benefit, then a one-tailed test is appropriate in order to increase the statistical power of detecting a beneficial effect.

Documentation of efficacy in human clinical trials is essential to the approval of new drugs in medical oncology, and REACT was specifically designed to examine the potential benefit of administering celecoxib among patients with early-stage breast cancer. Evidence from recent human clinical trials supports the use of targeted therapies for five molecular subtypes of breast cancer based on the presence/absence of estrogen receptors (ERs) and progesterone receptors (PRs) and ERRB-2 (HER2) expression (6). Celecoxib selectively inhibits the expression of COX-2, thereby interrupting multiple molecular events related to mammary carcinogenesis (7). In REACT, all patients had tumors negative for ERRB-2 expression, and 73% of the treated patients had tumors that were ER and PR positive. Regarding the examination of treatment effects in prespecified subgroups including patients who did not receive any chemotherapy, the investigators stated that “no evidence of differential treatment effects in any subgroup was observed for disease-free survival (1)”.

Although randomized clinical trials are technically experimental studies, random assignment of treatment, together with statistical adjustment for treatment–control differences, may not necessarily make the experimental and control groups identical. Rather, clinical trial findings must be interpreted in terms of the magnitude of the effect measure (HR) and the proximity of the *P*-value to 0.05, as well as evidence from the previous literature. The results of REACT are particularly important for women whose breast tumors are not treated by chemotherapy in that the trial “did not find any evidence that celecoxib treatment was associated with an increase in cardiac events compared with placebo”.

Cohesive scientific evidence from molecular, animal, and human investigations supports the hypothesis that overexpression of cyclooxygenase-2 drives mammary carcinogenesis and, reciprocally, that blockade of the inflammatory cyclooxygenase–prostaglandin cascade has strong potential for breast cancer prevention and therapy (2). The results of REACT are consistent with this hypothesis and support the continued testing of celecoxib for breast cancer patients not treated with chemotherapy.

## Conclusion

In REACT, celecoxib treatment (400 mg daily for 2 years) in a subgroup of 655 breast cancer patients who did not receive chemotherapy reduced the hazard ratio for recurrence over 10 years by 35% (HR = 0.65, 95% confidence interval 0.41–1.04, *P* = 0.035 by a one-tailed test of significance). These results underscore the importance of further testing of celecoxib in patients with early-stage breast cancer who are not treated by chemotherapy.

## Author contributions

RH wrote the initial manuscript, JS contributed to the methods and interpretation of findings. All authors contributed to the article and approved the submitted version.

## Conflict of interest

The authors declare that the research was conducted in the absence of any commercial or financial relationships that could be construed as a potential conflict of interest.

## Publisher’s note

All claims expressed in this article are solely those of the authors and do not necessarily represent those of their affiliated organizations, or those of the publisher, the editors and the reviewers. Any product that may be evaluated in this article, or claim that may be made by its manufacturer, is not guaranteed or endorsed by the publisher.
